# Evolution of Physical Demands of Australian Football League Matches from 2005 to 2017: A Systematic Review and Meta-Regression

**DOI:** 10.1186/s40798-021-00301-1

**Published:** 2021-04-28

**Authors:** Samuel J. Janetzki, Pitre C. Bourdon, Kevin I. Norton, Jackson C. Lane, Clint R. Bellenger

**Affiliations:** 1 Alliance for Research in Exercise, Nutrition and Activity (ARENA), University of South Australia, GPO Box 2471, Adelaide, South Australia 5001 Australia; 2 South Australian Sports Institute, Adelaide, South Australia Australia

**Keywords:** Australian Football League (AFL), global positioning system (GPS), match demands

## Abstract

**Background:**

There is extensive research investigating the match demands of players in the Australian Football League (AFL).

**Objective:**

This systematic literature review and meta-regression sought to analyse the evolution of in-game demands in AFL matches from 2005 to 2017, focusing on the relationship between volume and intensity.

**Methods:**

A systematic search of Ovid MEDLINE, Embase, Emcare, Scopus, SPORTDiscus, and Cochrane Library databases was conducted. Included studies examined the physical demands of AFL matches utilising global positioning system (GPS) technology. Meta-regression analysed the shift in reported volume (total distance and total match time) and intensity (metres per minute [m.min^−1^], sprint duration and acceleration) metrics for overall changes, across quarters and positional groups (forwards, nomadics and defenders) from 2005 to 2017 inclusive and for each year between 2005 and 2007, 2007 and 2010, 2010 and 2012, and 2012 and 2015/2017 breakpoints.

**Results:**

Distance (*p* = 0.094), m.min^−1^ (*p* = 0.494), match time (*p* = 0.591), time over 18 km·h^−1^ (*p =* 0.271), and number of accelerations greater than 4 km·h^−1^ (*p =* 0.498) and 10 km·h^−1^ (*p =* 0.335) in 1 s did not change from 2005 to 2017. From 2005 to 2007 volume decreased (− 6.10 min of match time; *p* = 0.010) and intensity increased (6.8 m.min^−1^ increase; *p* = 0.023). Volume and intensity increased from 2007 to 2010, evidenced by increases in total distance (302 m; *p* = 0.039), time over 18 km·h^−1^ (0.31 min; *p* = 0.005), and number of accelerations greater than 4 km·h^−1^ (41.1; *p* = 0.004) and 10 km·h^−1^ (3.6; *p* = 0.005) in 1 s. From 2010 to 2012, intensity decreased, evidenced by reductions in metres per minute (− 4.3; *p* = 0.022), time over 18 km·h^−1^ (− 0.93 min; *p* < 0.001), and number of accelerations greater than 4 km·h^−1^ (− 104.4; *p* < 0.001) and 10 km·h^−1^ (− 8.3; *p* < 0.001) in 1 s, whilst volume stabilised with no changes in distance (*p* = 0.068) and match time (*p* = 0.443). From 2012 to 2015/2017 volume remained stable and intensity increased with time over 18 km·h^−1^ (0.27 min; *p =* 0.008) and number of accelerations greater than 4 km·h^−1^ (31.6; *p* = 0.016) in 1 s increasing.

**Conclusions:**

Changes in volume and intensity of AFL match demands are defined by discrete periods from 2007 to 2010 and 2010 to 2012. The interaction of rule and interpretation changes and coaching strategies play a major role in these evolutionary changes. In turn, modified game styles impact player game demands, training, and selection priorities. Standardisation and uniformity of GPS data reporting is recommended due to inconsistencies in the literature.

## Key Points

- Changes in the volume and intensity of AFL match demands from 2005 to 2017 are defined by discrete, evolutionary periods from 2007 to 2010 and 2010 to 2012. Forwards exhibited the greatest change in volume and intensity of any positional group across these periods.
- Rule changes, professionalism of the sport and players, and the evolution of game style are postulated as the driving factors behind changes to the match demands of elite AFL players.
- Standardisation and uniformity are recommended for the manner and form in which AFL GPS match data are reported, particularly in relation to high-speed running metrics.

## Introduction

Australian rules football (AF) is a contact team sport played between two teams of 22 players, with 18 players permitted on the field at any one time and four players on the interchange bench. Teams can utilise up to 90 player interchanges per match. The aim of competing teams is to score more points than the opposition team over four, 20-minute quarters of match play plus time on [1]. The playing surface area varies considerably between grounds; however, the playing surface of the Melbourne Cricket Ground is approximately 80% larger than the biggest international standard soccer pitch. At the end of the first and third quarters, players are afforded a 6-minute rest period, with a 20-minute rest period at half-time. Currently in 2020, 18 professional teams compete in the Australian Football League (AFL), the premier AF competition.

AF is regarded as a physically and technically demanding sport [2–5]. The physical demands of an AF match vary considerably between playing positions [6], with global positioning system (GPS) analysis revealing players typically cover 11,000 to 17,000 metres during a match [7–9]. Historically, AF players were estimated to spend 60 to 90% of a match engaging in low intensity activities (e.g. walking and jogging), largely dependent on playing position and the method of player analysis utilised [10–12]. However, like most field-based team sports, AF has evolved over time [13, 14] with improvements in player athleticism, club, staff and player professionalism, and rule changes contributing significantly to the evolution of elite AF [14, 15]. In comparison to their late 1990 counterparts, modern day AF players are required to engage in more frequent, high intensity sprint efforts for longer periods of time [7], with the ability to recover from these intermittent, high intensity activities being a defining characteristic of the modern, elite AF player [16].

Professional sport’s development more broadly has been characterised by the rapid evolution of game intensity and player demands [17–21]. With elite AF being a multi-billion-dollar industry and the players being the primary asset, it is essential AFL clubs have a comprehensive understanding of individual player and team movement patterns, with the goal of maximising player and team performance [14].

The introduction of GPS technology to the AFL in 2005 provided clubs with the ability to more accurately monitor and understand their players’ in-game movement patterns [7]. Quantifying game trends aids the optimisation of player preparation for matches by informing training drills and developing fitness profiles suited to high-performance AF, as well as help predict future game demands and assist in player selection. This led to significant growth in the literature investigating the match demands of AFL players [7, 22, 23]. Studies have more recently examined the relationship between in-game running demands on individual and team match performance [24], as well as the influence of individual player characteristics such as player calibre [24, 25], experience [26], fitness [27] and the number of interchange rotations [28, 29] on match running performance. There has also been consideration of match-related factors on physical output such as team success [24] and ladder position [30], opposition strength [31], number of stoppages [31], match location (home or away) [31] and time of match during the season [4, 31].

Measuring speed and acceleration data are important to understand the demands of field-sport athletes like AF players [32]. Studies analysing the application of GPS technology in AFL matches suggest there are many factors attributable to the variation in match activity profiles of elite AF players. Importantly, recent improvements in the validity and reliability of GPS units [33–35] permits greater precision of the match analysis data [36], however the reliability of movement data collected at speeds in excess of 20 km·h^-1^ remains in question [33].

Despite the myriad of studies which have quantified and investigated the match demands of elite AF, there is minimal longitudinal evidence supporting the anecdotal increase in the intensity of modern AF matches. Given the perceived shift in game demands of AFL matches over the last 15 years, the aim of this systematic literature review was to specifically quantify any shifts in AFL match movement profiles by analysing and describing published results from GPS volume (total distance and total match time) and intensity (metres per minute [m.min^-1^], time spent at speeds over 18 km·h^-1^ and the number of accelerations greater than 4 km·h^-1^ and 10 km·h^-1^ in one second) metrics from AFL matches.

## Methods

This review followed the Preferred Reporting Items for Systematic Reviews and Meta-Analyses (PRISMA) statement for improved reporting of systematic reviews [37].

### Literature search

A systematic search of the literature was conducted on the 4th of December 2019 in the following databases: Ovid MEDLINE, Embase, Emcare, Scopus, SPORTDiscus, and Cochrane Library. Database update alerts were monitored until August 2020 for any additional articles that met the inclusion criteria. Database searches were complemented with pearling of the reference lists of relevant studies.

Title, abstract, and keyword searches were conducted with the following search strategy:

1. Football/ AND Australia/

AND

2. Australian football* OR Australian rules football* OR Australian football league OR Australian rules

AND

3. geographic information systems/ OR task performance and analysis/ OR physical fitness/ OR acceleration/ OR physical conditioning, human/ OR endurance training/ OR high-intensity interval training/ OR exp running/ OR GPS or global position* system? or geographic* information system? or geographic* position* system? or match demand* or game demand* or running demand* or activity profil* or total distance? or HSR or high speed running or high-speed running or sprint* or duration or meter* per minute* or metre* per minute* or meter?-per-minute? or metre?-per-minute? or physical* condition* or endurance or high-intensity interval train* or accelerat* or fitness

### Eligibility criteria

Studies were eligible for inclusion in this review if their participants were professional, elite male AFL players competing in premiership season and finals matches. Studies were included if they analysed the match running demands of participants utilising GPS technology and reported on any of the following metrics: total distance, high-speed running, m.min^-1^, accelerations, sprint and game duration. Studies which analysed training demands were included provided the training demands were analysed in conjunction with game demands. However, studies were excluded if game demand data could not be extracted independently of training data. Unpublished, non-English or qualitative studies were not eligible for inclusion in this review, except for eleven studies [38–48] which were official, non-peer reviewed reports generated for the AFL detailing GPS data obtained from premiership matches from the 2005 to 2015 seasons.

To be eligible for inclusion in the meta-regression, studies were required to report mean and standard deviations of the following GPS volume (total distance and game duration [total match time]) and intensity (m.min^-1^, time over 18 km·h^-1^ and accelerations greater than 4 km·h^-1^ and 10 km·h^-1^ in one second) metrics. Studies which reported ranges were excluded since calculations of an estimated standard deviation [49] to in turn calculate a standard error for the meta-regression, proved inaccurate. Studies which failed to report the number of GPS files analysed (to determine the sample size) were included provided the mean and standard deviation were reported.

### Study selection

Studies identified in the systematic search were included for narrative review, with eligible studies included in the meta-regression. Articles uncovered from the search were exported into a reference management software program (Endnote version X8.2, Thomson Reuters, 2012). All references were then imported into Covidence (Covidence Systematic Review Software, Veritas Health Innovation, 2013) where all duplicates were removed. The eligibility of studies was assessed in Covidence independently by two investigators (SJJ and JCL), with conflicts resolved by consensus. All studies were screened initially by title and abstract against the eligibility criteria to exclude irrelevant studies. The remaining studies were assessed for full-text eligibility using the eligibility criteria. For studies which appeared to analyse and report on GPS match data but did not report the basic summaries of the GPS data or the season(s) analysed in the paper, corresponding authors were emailed to obtain the underlying data and seek clarification if required. If no response was received, those articles were also excluded. Where articles analysed the same data set, only the article which reported the larger sample size (*n*) was included.

Data extraction was conducted by the lead author (SJJ) and confirmed by a second investigator (JCL). The following information was obtained from the included studies: publication details (year, author[s], country), participant characteristics (number of participants, age, body mass, height), the season(s) analysed, GPS unit specifications (Hz, manufacturer), and location of AFL club, if reported. All data relating to the following GPS metrics were extracted: total distance, high-speed running, accelerations (greater than 4 km·h^−1^ and 10 km·h^−1^ in 1 s), time over 18 km·h^−1^, m.min^−1^, sprint duration, and game duration. If reported, data from GPS metrics were further categorised into quarters and the following positional groups: forwards, nomadic (midfielders and ruckmen), and defenders.

### Risk of bias assessment

The Cochrane Collaboration tool was used by the lead author (SJJ) and confirmed by a second investigator (JCL) to assess risk of bias [50]. The tool was used to assess selection, performance, detection, attrition and reporting bias from the studies identified from the systematic search.

### Statistical analysis

Linear regression was performed on all variables in Stata 16.0 (College Station, Texas) to determine the seasonal change in variables from the 2005 to 2017 AFL seasons. To weight each data point across a year, the standard error was calculated by dividing the standard deviation by the square-root of the sample size (number of GPS files analysed). Data were presented as mean ± 95% confidence intervals with statistical significance set at *p* < 0.05.

Meta-regressions were conducted on all variables to assess the linear relationship between selected breakpoints from 2005 to 2007, 2007 to 2010, 2010 to 2012, and 2012 to 2015 or 2017 (depending on the final year in which data were recorded) to assess the change in variables and selected GPS metrics of interest across seasons. These breakpoints were selected using *adjusted R-squared* values to assess the goodness of fit of the data and required the analysis of a minimum of three seasons’ data. These periods were chosen as they exhibited consistent changes across all metrics. To ensure uniformity in the units of reported data, total distance data were converted to metres and metres per minute data were converted to metres per second (m·s^−1^). Data points were weighted using the standard error.

## Results

**Fig. 1:**
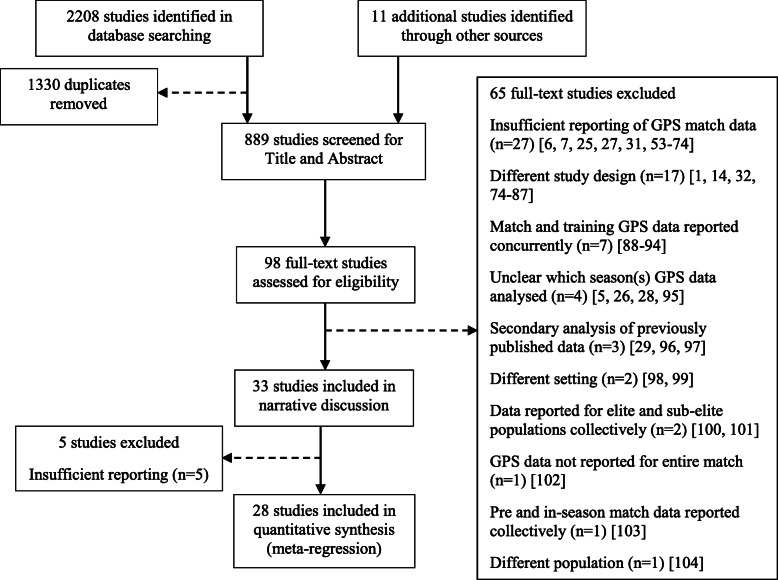
Flowchart of literature search. *GPS*, global positioning system; *n*, number of studies

The initial search identified 2208 studies, with 11 studies identified through other sources. 1330 studies were removed as duplicates, 791 studies were identified by title and abstract as irrelevant, and a further 65 studies were not relevant to this review. A summary of the search detailing the number of studies included in the narrative discussion and meta-regression is shown in Fig. 1.

### Reasons for exclusion

A total of 65 studies [1, 5–7, 14, 25–29, 31, 32, 51–102], of which the full text was reviewed, were excluded from the narrative discussion. The reasons for exclusion from the narrative discussion are detailed in Figure 1. Of note, studies with a different study design were either systematic reviews or used methodology inconsistent with the inclusion criteria of this review. Studies with a different setting reported on data from a time course prior to the period of interest and the study with a different population reported results from a different sport and population of interest.

Five studies were included for the narrative discussion but were excluded from the meta-regression as they failed to report a mean and/or standard deviation [3, 4, 103, 104] and reported m.min^-1^ data per rotation within quarters [9]. No response was provided by these authors who were emailed for the requisite information.

### Risk of bias

Random sequence generation and allocation concealment selection bias were assessed as unclear in all studies, as the nature of the included studies did not articulate how and from which AFL clubs the participants were selected.

Performance and detection bias, as a result of participant and study personnel blinding of the outcome assessment, were not assessed in this review. The nature of the identified studies included did not provide enough detail to assess these biases.

Attrition bias within the identified studies was assessed as either low risk or unclear. Eight studies were assessed at high risk of a reporting bias [4, 8, 9, 36, 103–106]. Five studies analysed GPS data collected over multiple seasons, but only reported a single mean value encompassing all the seasons analysed [4, 8, 36, 104, 105]. Two studies collected and analysed GPS data relevant to this review but failed to report the overall mean for the variables analysed [9, 106], whilst Corbett et al. [104] reported the mean value for some but not all of the GPS variables analysed. Colby et al. [103] reported predicted match data using individual player averages, where players did not wear a GPS unit, participate in the match or the GPS signal was deemed unreliable. Three studies were originally assessed at a high risk of reporting bias, however the provision of results of the mean values of the GPS data analysed by the authors, subsequently rendered these papers with a low risk [107–109].

### Narrative discussion

A total of 33 studies [2–4, 8, 9, 17, 22, 24, 36, 38–48, 103–115] reported on GPS metrics from AFL matches across the 2005 to 2017 seasons. All data extracted from these studies are summarised in Table 1, highlighting the inconsistency of reporting on GPS metrics in the literature. Within these studies, 27 reported on m.min^-1^ [2, 4, 8, 9, 17, 22, 24, 36, 38–48, 104, 107–111, 113, 114], 25 reported on total distance [2, 4, 8, 22, 24, 36, 38–48, 103, 107–112, 115], 22 reported on total match time [8, 17, 22, 24, 36, 38–48, 108–112, 115], 12 reported on accelerations greater than 4 km·h^-1^ and 10 km·h^-1^ in one second [22, 38–48] and 11 reported on time over 18 km·h^-1^ [38–48].

**Table 1:** Data extraction from all studies included in the descriptive synthesis

	2005	2006	2007	2008	2009	2010	2011	2012	2013	2014	2015	2016	2017
**VOLUME METRICS**													
**TD – match (m)**	12500 ± 1700 [22] 12450 ± 1650 [40–42, 46]	12500 ± 1700 [22] 12510 ± 1710 [41, 42, 46]	12400 ± 1600 [22] 12420 ± 1580 [42, 46]	12734 ± 1596 [110] 12311 ± 1729 [111] 12620 ± 1872 [8] 12200 ± 1900 [22] 12240 ± 2010 [46–48]	12734 ± 1596 [110] 13455 ± 1764 [24] 12620 ± 1872 [8] 13190 ± 2040 [43, 47, 48]	12620 ± 1872 [8] 13040 ± 2010 [43, 44, 48]	13556 ± 1206 [36] 13430 ± 1990 [38, 43–45]	13556 ± 1206 [36] 13399 ± 69; 13399 (13150-13644) [103] 11954 ± 1259; 11954 (7572-14948) [115] 12360 ± 1920 [38, 44, 45]	12859 ± 1529 [2] 11950 ± 2056 [109] 12400 ± 1770 [38, 39, 45]	12820 ± 1750 [38, 39]	13200 ± 1680 [39]	11608 ± 3573 [112] 13048 ± 929 [108]	13257 ± 1778 [107] 13233 ± 915 [108]
**TD – halves (m)**				1^st^ – 6229 ± 972 2^nd^ – 6082 ± 1080 [111]									
**TD – start, middle, end season (m)**							S – 12948 (12775-13120) M – 13199 (13001-13397) E – 13193 (12988-13398) [4]	S – 12948 (12775-13120) M – 13199 (13001-13397) E – 13193 (12988-13398) [4]					
**Match time – (min)**	111.01 ± 13.50 [22, 40–42, 46]	111.51 ± 14.13 [22, 41, 42, 46]	104.19 ± 12.16 [22] 104.19 ± 12.16 [42, 46]	101.91 ± 9.80 [110] 98.00 ± 14.00 [111] 98.40 ± 11.60 [8] 100.10 ± 14.22 [22] 100.27 ± 15.14 [46–48]	101.91 ± 9.80 [110] 93.55 ± 10.30 [17] 100.50 ± 14.30 [24] 98.40 ± 11.60 [8] 106.56 ± 14.45 [43, 47, 48]	98.40 ± 11.60 [8] 105.14 ± 14.38 [43, 44, 48]	104.90 ± 10.00 [36] 109.53 ± 14.47 [38, 43–45]	104.90 ± 10.00 [36] 101.00 ± 11.00 [115] 104.10 ± 13.42 [38, 44, 45]	99.00 ± 19.00 [109] 103.18 ± 12.14 [38, 39, 45]	106.58 ± 12.46 [38] 106.35 ± 12.28 [39]	109.07 ± 12.35 [39]	87.80 ± 27.20 [112] 100.90 ± 7.60 [108]	100.30 ± 7.10 [108]
**INTENSITY METRICS**													
**m.min**^**-1**^ **– match**	113.3 ± 10.0 [22] 112.7 ± 11.2 [40–42, 46]	113.3 ± 15.0 [22] 113.0 ± 15.7 [41, 42, 46]	120.0 ± 13.3 [22] 119.7 ± 13.5 [42, 46]	127.0 ± 17.0 [110] 128.0 ± 12.0 [111] 129.0 ± 17.0 [8] 130.0 ± 18.0 [114] 121.7 ± 11.7 [22] 122.2 ± 10.7 [46–48]	127.0 ± 17.0 [110] 134.0 ± 12.1 [17] 134.7 ± 12.4 [24] 129.0 ± 17.0 [8] 140.0 ± 15.0 [114] 123.7 ± 10.7 [43, 47, 48]	129.0 ± 17.0 [8] 124.7 ± 10.5 [43, 44, 48]	129.6 ± 9.5 [36] 122.5 ± 10.7 [38, 43–45]	129.6 ± 9.5 [36] 118.7 ± 10.7 [38, 44, 45]	123.0 ± 12.0 [2] 121.7 ± 14.1 [109] 120.3 ± 11.3 [38, 39, 45]	130.5 [104] 131.8 ± 9.1 [113] 119.7 ± 10.3 [38, 39]	130.5 [104] 120.5 ± 9.8 [39]	128.0 ± 7.4 [108]	124.4 ± 4 [107] 130.7 ± 7.1 [108]
**m.min**^**-1**^ **– halves**				1^st^ – 131.0 ± 13.0 2^nd^– 125.0 ± 14.0 [111]									
**m.min**^**-1**^ **– rotations per quarter**				Q1– r1: 137.9 ± 17.2 r2: 137.0 ± 12.5 Q2 – r1: 136.2 ± 10.8 r2: 123.2 ± 16.7 Q3 – r1: 119.0 ± 16.0 r2: 128.0 ± 8.2 Q4 – r1: 121.6 ± 13.1 r2: 125.4 ± 13.6 [9]									
**m.min**^**-1**^ **– rotations per quarter: finals**				Q1– r1: 152.7 ± 17.8 r2: 145.6 ± 22.8 Q2 – r1: 149.3 ± 26.0 r2: 139.0 ± 52.0 Q3 – r1: 130.6 ± 33.7 r2: 146.3 ± 9.7 Q4 – r1: 149.2 ± 13.7 r2: 133.3 ± 32.5 [9]									
**m.min**^**-1**^ **– start, middle, end season**							S – 123.0 (121.0-125.0) M – 125.0 (123.0-127.0) E – 124.0 (122.0-127.0) [4]	S – 123.0 (121.0-125.0) M – 125.0 (123.0-127.0) E – 124.0 (122.0-127.0) [4]					
**Accelerations**													
**Number of accelerations – 2.78 - 10** **m**·**s**^**-2**^				96.0 ± 41.0 [110] 82.0 ± 26.0 [8]	96.0 ± 41.0 [110] 82.0 ± 26.0 [8]	82.0 ± 26.0 [8]							
**Number of accelerations per minute – ≥2.78** **m**·**s**^**-2**^				1.0 ± 0.4 [110] 0.8 ± 0.3 [8] 1.1 ± 0.5 [114]	1.0 ± 0.4 [110] 0.8 ± 0.3 [8] 1.2 ± 0.5 [114]	0.8 ± 0.3 [8]	0.5 ± 0.1 [36]	0.5 ± 0.1 [36]					
**Number of accelerations – 3 - 15** **m**·**s**^**-2**^								29.0 ± 8.0; 29.0 (8-53) [115]					
**Number of accelerations >4 km**^**.**^**h**^**-1**^ **in 1 second**	240.8 ± 43.2 [22, 40–42, 46]	251.0 ± 50.0 [22] 251.2 ± 50.1 [41, 42, 46]	253.0 ± 41.0 [22] 253.0 ± 41.1 [42, 46]	246.0 ± 47.0 [22] 292.0 ± 87.0 [46–48]	322.0 ± 135.0 [43, 47, 48]	402.0 ± 194.0 [43, 44, 48]	233.0 ± 119.0 [38, 43–45]	186.0 ± 43.0 [38, 44, 45]	188.0 ± 39.0 [38, 39, 45]	242.0 ± 60.0 [38, 39]	255.0 ± 56.0 [39]		
**Number of accelerations >10 km**^**.**^**h**^**-1**^ **in 1 second**	10.0 ± 4.4 [40–42, 46]	11.5 ± 6.0 [41, 42, 46]	12.2 ± 5.3 [42, 46]	14.5 ± 8.8 [46–48]	17.3 ± 14.4 [43, 47, 48]	25.4 ± 19.3 [43, 44, 48]	11.0 ± 11.5 [38, 43–45]	6.9 ± 5.1 [38, 44, 45]	6.9 ± 4.6 [38, 39, 45]	8.9 ± 5.8 [38, 39]	8.1 ± 4.8 [39]		
**Number of surges >18km**^**.**^**h**^**-1**^	89.0 ± 20.0 [22]	90.0 ± 24.0 [22]	93.0 ± 20.0 [22]	86.0 ± 2.0 [22]									
**Time per minute engaging in acceleration - 2.78 - 10** **m**·**s**^**-2**^ **(%)**							1.4 ± 0.4 [36]	1.4 ± 0.4 [36]					
**Sprints**													
**Number of sprints ≥20 km**^**.**^**h**^**-1**^ **– match**				74.0 ± 23.0 [111]									
**Number of sprints ≥20 km**^**.**^**h**^**-1**^ **– halves**				1^st^ – 39.0 ± 13.0 2^nd^ – 35.0 ± 12.0 [111]									
**Number of sprints ≥20 km**^**.**^**h**^**-1**^ **per minute – match**				0.8 ± 0.2 [111]	0.7 ± 0.2 [17] 0.7 ± 0.1 [24]								
**Number of sprints ≥20 km**^**.**^**h**^**-1**^ **per minute – halves**				1^st^ – 0.8 ± 0.3 2^nd^ – 0.7 ± 0.2 [111]									
**Time sprinting >20 km**^**.**^**h**^**-1**^ **– match (%)**					6.2 ± 1.8 [17]								
**Number of sprints ≥23 km**^**.**^**h**^**-1**^ **– quarters**	Q1 – 7.7 ± 2.6 Q2 – 7.2 ± 3.1 Q3 – 7.5 ± 4.7 Q4 – 6.2 ± 2.8 [105]	Q1 – 7.7 ± 2.6 Q2 – 7.2 ± 3.1 Q3 – 7.5 ± 4.7 Q4 – 6.2 ± 2.8 [105]	Q1 – 7.7 ± 2.6 Q2 – 7.2 ± 3.1 Q3 – 7.5 ± 4.7 Q4 – 6.2 ± 2.8 [105]										
**Number of sprints >23 km**^**.**^**h**^**-1**^ **– start, middle, end season**							S – 17.9 (16.9-18.9) M – 18.2 (17.1-19.3) E – 19.8 (18.7-21.0) [4]	S – 17.9 (16.9-18.9) M – 18.2 (17.1-19.3) E – 19.8 (18.7-21.0) [4]					
**Sprinting distance >23 km**^**.**^**h**^**-1**^ **– start, middle, end season (m)**							S– 405 (379-431) M – 431 (401-461) E – 459 (427-490) [4]	S– 405 (379-431) M – 431 (401-461) E – 459 (427-490) [4]					
**Number of sprints ≥ 24 km**^**.**^**h**^**-1**^ **– match**												11.8 ± 6.7 [108]	11.8 ± 5.9 [107] 12.3 ± 5.9 [108]
**Sprinting distance ≥24 km**^**.**^**h**^**-1**^ **– match (m)**												272.0 ± 163.9 [108]	243.2 ± 119.0 [107] 266.9 ± 139.3 [108]
**Time sprinting >24 km**^**.**^**h**^**-1**^**– match (s)**												36.0 ± 21.0 [108]	32.0 ± 17.0 [108]
**Sprinting distance ≥25.2 km**^**.**^**h**^**-1**^ **– match (m)**				328 ± 164 [8]	328 ± 164 [8]	328 ± 164 [8]		80 ± 55; 80 (0-253) [115]					
**Sprinting ≥25.2 km**^**.**^**h**^**-1**^ **– match (m.min**^**-1**^**)**				3.4 ± 1.7 [8]	3.4 ± 1.7 [8]	3.4 ± 1.7 [8]							
**Sprint distance >75% of individual’s maximum speed – match (m)**								268 (254-283) [103]					
**HSR / HIE**													
**Number of HSR ≥19.8 - 36 km**^**.**^**h**^**-1**^ **– match**				82.0 ± 21.0 [8]	82.0 ± 21.0 [8]	82.0 ± 21.0 [8]							
**Number of HSR ≥19.8 - 36 km**^**.**^**h**^**-1**^ **per min – match**				0.8 ± 0.2 [8]	0.8 ± 0.2 [8]	0.8 ± 0.2 [8]							
**Number of HSR ≥25.2 km**^**.**^**h**^**-1**^ **– match**				22.0 ± 9.0 [8]	22.0 ± 9.0 [8]	22.0 ± 9.0 [8]							
**Number of HSR ≥25.2 km**^**.**^**h**^**-1**^ **per min – match**				0.2 ± 0.1 [8]	0.2 ± 0.1 [8]	0.2 ± 0.1 [8]							
**HSR >4** **m**·**s**^**-1**^ **percentage of total distance (%)**							10.3 ± 2.2 [36]	10.3 ± 2.2 [36]		0.3 [104]	0.3 [104]		
**HSR >4** **m**·**s**^**-1**^ **mean number per min**					2.1 ± 0.4 [24]		1.6 ± 0.3 [36]	1.6 ± 0.3 [36]					
**Number of HIE ≥15 km**^**.**^**h**^**-1**^ **– match**				271.0 ± 57.0 [111]									
**Number of HIE ≥15 km**^**.**^**h**^**-1**^ **– halves**				1^st^ – 141.0 ± 31.0 2^nd^– 130.0 ± 33.0 [111]									
**TD HSR >4** **m**^**.**^**s**^**-1**^ **(14.4 km**^**.**^**h**^**-1**^**) – match (m)**									3678 ± 1144 [2]			3198 ± 1165 [112]	
**HSR >4** **m**^**.**^**s**^**-1**^ **(14.4 km**^**.**^**h**^**-1**^**) – match (m**.**min**^**-1**^**)**					30.3 ± 6.9 [24]		28.6 ± 6.3 [36]	28.6 ± 6.3 [36]	36.0 ± 10.0 [2]	33.5 [104] 36.4 ± 7.1 [113]	33.5 [104]	36.4 [112]	
**TD HSR >4** **m**·**s**^**-1**^ **(14.4 km**^**.**^**h**^**-1**^**) – quarters (m)**	Q1 – 1090 ± 212 Q2 – 980 ± 219 Q3 – 971 ± 256 Q4 – 844 ± 198 [105]	Q1 – 1090 ± 212 Q2 – 980 ± 219 Q3 – 971 ± 256 Q4 – 844 ± 198 [105]	Q1 – 1090 ± 212 Q2 – 980 ± 219 Q3 – 971 ± 256 Q4 – 844 ± 198 [105]										
**TD HSR >4** **m**·**s**^**-1**^ **(14.4 km**^**.**^**h**^**-1**^**) – start, middle, end season (m)**							S – 3462 (3336-3589) M – 3492 (3347-3638) E – 3696 (3352-3840) [4]	S – 3462 (3336-3589) M – 3492 (3347-3638) E – 3696 (3352-3840) [4]					
**TD HSR 4.17 - 10** **m**·**s**^**-1**^ **(15.01 - 36 km**^**.**^**h**^**-1**^**) – match (m)**				3334 ± 756 [110]	3334 ± 756 [110]								
**HSR 4.17 - 10** **m**·**s**^**-1**^ **(15.01 - 36 km**^**.**^**h**^**-1**^**) – match (m.min**^**-1**^**)**				34.0 ± 9.0 [110] 34.9 ± 8.8 [114]	34.0 ± 9.0 [110] 38.1 ± 8.8 [114]								
**HSR 4.17 - 10** **m**·**s**^**-1**^ **(15.01 - 36 km**^**.**^**h**^**-1**^**) – quarter rotations: (m.min**^**-1**^**)**				Q1– r1: 37.5 ± 12.5 r2: 37.5 ± 9.4 Q2 – r1: 43.2 ± 18.9 r2: 32.8 ± 10.4 Q3 – r1: 32.1 ± 10.0 r2: 37.1 ± 7.9 Q4 – r1: 32.8 ± 9.6 r2: 37.5 ± 13.9 [9]									
**HSR 4.17 - 10** **m**·**s**^**-1**^ **(15.01 - 36 km**^**.**^**h**^**-1**^**) – quarter rotations: finals (m.min**^**-1**^**)**				Q1 – r1: 43.2 ± 13.9 r2: 40 ± 13.5 Q2 – r1: 42.8 ± 16.4 r2: 39.2 ± 15.8 Q3 – r1: 35.3 ± 10.0 r2: 40.0 ± 17.5 Q4 – r1: 40.7 ± 10.7 r2: 33.5 ± 10.7 [9]									
**TD HSR >18 km**^**.**^**h**^**-1**^ **– match (m)**												1888 ± 421 [108]	1657 ± 470 [107] 1938 ± 430 [108]
**HSR >18 km**^**.**^**h**^**-1**^ **– match (m.min**^**-1**^**)**												18.9 ± 4.6 [108]	13.1 ± 3.8 [107] 19.1 ± 4.6 [108]
**Time over 18 km**^**.**^**h**^**-1**^ **– match (min)**	5.24 ± 1.31 [40–42, 46]	5.25 ± 1.49 [41, 42, 46]	5.39 ± 1.28 [42]	5.40 ± 1.40 [47, 48]	6.18 ± 1.54 [43, 47, 48]	6.20 ± 1.51 [43, 44, 48]	5.25 ± 2.15 [38, 43–45]	4.45 ± 1.36 [38, 44, 45]	4.53 ±1.38 [38, 39, 45]	5.07 ± 1.72 [38, 39]	5.16 ± 1.40 [39]		
**HSR 19.1 - 24 km**^**.**^**h**^**-1**^ **– match (m.min**^**-1**^**)**										11.7 ± 2.5 [113]			
**TD HSR >19.9 km**^**.**^**h**^**-1**^ **– start, middle, end season (m)**							S – 1023 (977-1070) M – 1056 (1003-1109) E – 1145 (1089-1200) [4]	S – 1023 (977-1070) M – 1056 (1003-1109) E – 1145 (1089-1200) [4]					
**HSR >20 km**^**.**^**h**^**-1**^ **– match (m.min**^**-1**^**)**					15.1 ± 4.4 [24]								
**TD HSR >19.9 km**^**.**^**h**^**-1**^ **– start, middle, end season (m)**							S – 405 (379-431) M – 431 (401-461) E – 459 (427-490) [4]	S – 405 (379-431) M – 431 (401-461) E – 459 (427-490) [4]					
**TD HSR >24 km**^**.**^**h**^**-1**^ **– start, middle, end season (m)**													243 ± 119 [107]
**Number of HSR >24 km**^**.**^**h**^**-1**^ **efforts**													11.8 ± 5.9 [107]
**HSR >24 km**^**.**^**h**^**-1**^ **– match (m.min**^**-1**^**)**										2.3 ± 0.8 [113]			
**TD HSR >25 km**^**.**^**h**^**-1**^ **– start, middle, end season (m)**												154 ± 105 [112]	

*HIR* high-intensity running, *HIE* high-intensity efforts, *m* metres, *m.min*^*-1*^ metres per minute, *min* minutes, *m*·*s*^*-1*^ meters per second, *s* seconds, *TD* total distance,

*±* mean and standard deviation, *(-)* mean and range, *%* percentage

Table 2 summarises the data extracted from all positional groups combined, in addition to position specific summaries for forwards, nomadics, and defenders, along with data from each quarter.

**Table 2:** All data extracted from studies included in the descriptive synthesis reporting positional and quarter data

		2005	2006	2007	2008	2009	2010	2011	2012	2013	2014	2015
**FWD**	**m.min**^**-1**^	108.7 ± 11.2 [40]	108.3 ± 14.5 [41]	106.7 ± 10.2 [42]	114.0 ± 10.2 [46]	116.0 ± 11.7 [47]	114.8 ± 13.5 [43, 48]	113.8 ± 8.7 [43]	111.3 ± 10.0 [44]	112.2 ± 10.8 [45]; 114.8 ± 15.0 [109]	112.5 ± 10.0 [38]	113.2 ± 10.0 [39]
	**TO**	4.43 ± 1.30 [40]	4.94 ± 1.41 [41]	4.36 ± 1.14 [42]	4.52 ± 1.24 [46]	5.08 ± 1.33 [47]	5.09 ± 1.40 [43, 48]	4.18 ± 1.08 [43]	4.00 ± 1.22 [44]	4.06 ± 1.20 [45]	4.34 ± 1.46 [38]	4.23 ± 1.18 [39]
	**TD**	11905 ± 1930 [40]	12170 ± 1600 [41]	11660 ± 1508 [42]	11920 ± 2080 [46]	12850 ± 2100 [47]	12500 ± 2160 [43, 48]	12890 ± 1890 [43]	12020 ± 1950 [44]	11970 ± 1870 [45]; 11722 ± 1182 [109]	12310 ± 1940 [38]	12790 ± 1730 [39]
	**TMT**	110.36 ± 15.08 [40]	113.41 ± 14.36 [41]	109.48 ± 11.97 [42]	104.28 ± 14.41 [46]	110.37 ± 13.19 [47]	108.56 ± 14.55 [43, 48]	113.25 ± 14.50 [43]	107.53 ± 13.40 [44]	106.43 ± 12.34 [45]; 103.00 ± 12.00 [109]	108.69 ± 13.93 [38]	112.25 ± 12.15 [39]
**NOM**	**m.min**^**-1**^	116.3 ± 12.5 [40]	-	121.5 ± 12.7 [42]	123.8 ± 9.8 [46]	124.7 ± 10.3 [47]	125.3 ± 9.7 [43, 48]	136.0 ± 11.0 [106]; 123.8 ± 10.3 [43]	120.36 ± 10.2 [44]	122.5 ± 10.5 [45]; 123.0 ± 14.2 [109]	121.8 ± 9.5 [38]; 141.2 ± 7.0 [113]	122.0 ± 9.2 [39]
	**TO**	5.51 ± 2.02 [40]	-	5.49 ± 1.25 [42]	5.50 ± 1.38 [46]	6.26 ± 1.55 [47]	6.29 ± 1.49 [43, 48]	5.35 ± 2.19 [43]	4.53 ± 1.37 [44]	5.06 ± 1.40 [45]	5.30 ± 1.74 [38]	5.27 ± 1.41 [39]
	**TD**	12930 ± 3700 [40]	-	12520 ± 1570 [42]	12310 ± 2010 [46]	13230 ± 2060 [47]	13080 ± 2000 [43, 48]	13460 ± 2000 [43]	12390 ± 1890 [44]	12470 ± 1760 [45]; 11967 ± 2207 [109]	12930 ± 1710 [38]	13250 ± 1608 [39]
	**TMT**	111.48 ± 32.12 [40]	-	103.58 ± 12.17 [42]	99.34 ± 15.11 [46]	106.20 ± 14.53 [47]	104.28 ± 14.28 [43, 48]	108.52 ± 14.36 [43]	103.15 ± 12.52 [44]	98 ± 17 [109]; 101.52 ± 11.4 [45]	105.48 ± 11.93 [38]	108.05 ± 12.24 [39]
**DEF**	**m.min**^**-1**^	110.7 ± 11.2 [40]	100.8 ± 11.8 [41]	106.0 ± 13.8 [42]	113.5 ± 10.2 [46]	120.5 ± 9.5 [47]	115.5 ± 9.7 [43, 48]	116.2 ± 10.2 [43]	110.2 ± 9.5 [44]	111.0 ± 10.2 [45]; 120.8 ± 8.6 [109]	111.8 ± 8.8 [38]	113.0 ± 8.3 [39]
	**TO**	5.17 ± 1.31 [40]	4.17 ± 1.15 [41]	4.19 ± 1.14 [42]	4.35 ± 1.27 [46]	6.04 ± 1.45 [47]	5.17 ± 1.36 [43, 48]	4.20 ± 1.44 [43]	3.55 ± 1.14 [44]	3.57 ± 1.07 [45]	4.20 ± 1.22 [38]	4.18 ± 1.06 [39]
	**TD**	12120 ± 2130 [40]	11650 ± 1290 [41]	11660 ± 1170 [42]	11880 ± 1920 [46]	13150 ± 1810 [47]	13080 ± 2000 [43, 48]	13500 ± 1880 [43]	12240 ± 2190 [44]	12280 ± 1760 [45]; 12129 ± 1768 [109]	12590 ± 1720 [38]	13130 ± 1620 [39]
	**TMT**	110.18 ± 19.40 [40]	116.33 ± 11.73 [41]	110.08 ± 11.34 [42]	104.49 ± 14.49 [46]	109.19 ± 13.56 [47]	113.58 ± 13.52 [43, 48]	116.37 ± 14.50 [43]	110.45 ± 15.37 [44]	110.45 ± 12.35 [45]; 101.00 ± 18.00 [109]	111.76 ± 12.93 [38]	115.28 ± 12.06 [39]
**Q1**	**m.min**^**-1**^	117.0 ± 14.0 [105]	117.0 ± 14.0 [105]	117.0 ± 14.0 [105]	127.7 ± 14.2 [46]	128.7 ± 13.7 [47]	129.0 ± 12.8 [43, 48]	124.5 ± 13.0 [43]	123.0 ± 12.8 [44]	124.8 ± 13.3 [45]	124.2 ± 12.7 [38]	126.2 ± 11.8 [39]
	**TD**	3463 ± 403 [105]	3463 ± 403 [105]	3463 ± 403 [105]	3070 ± 630 [46]	3300 ± 640 [47]	3350 ± 590 [43, 48]	3270 ± 570 [43]	3180 ± 600 [44]	3260 ± 540 [45]	3320 ± 530 [38]	3480 ± 490 [39]
**Q2**	**m.min**^**-1**^	108.0 ± 15.0 [105]	108.0 ± 15.0 [105]	108.0 ± 15.0 [105]	122.8 ± 12.8 [46]	124.8 ± 12.8 [47]	125.5 ± 12.8 [43, 48]	120.8 ± 12.7 [43]	119.0 ± 12.5 [44]	121.2 ± 13.2 [45]	120.3 ± 11.8 [38]	121.0 ± 11.0 [39]
	**TD**	3186 ± 461 [105]	3186 ± 461 [105]	3186 ± 461 [105]	2930 ± 660 [46]	3180 ± 610 [47]	3180 ± 640 [43, 48]	3090 ± 640 [43]	3100 ± 600 [44]	3140 ± 520 [45]	3300 ± 560 [38]	3390 ± 550 [39]
**Q3**	**m.min**^**-1**^	108.0 ± 17.0 [105]	108.0 ± 17.0 [105]	108.0 ± 17.0 [105]	122.2 ± 13.3 [46]	123.5 ± 13.3 [47]	125.0 ± 13.2 [43, 48]	120.7 ± 12.3 [43]	118.2 ± 12.7 [44]	119.3 ± 13.7 [45]	119.0 ± 12.8 [38]	119.7 ± 11.8 [39]
	**TD**	3232 ± 460 [105]	3232 ± 460 [105]	3232 ± 460 [105]	2860 ± 670 [46]	3070 ± 630 [47]	3090 ± 630 [43, 48]	3000 ± 630 [43]	3050 ± 600 [44]	3060 ± 570 [45]	3050 ± 600 [38]	3100 ± 600 [39]
**Q4**	**m.min**^**-1**^	103.0 ± 14.0 [105]	103.0 ± 14.0 [105]	103.0 ± 14.0 [105]	118.3 ± 13.3 [46]	119.5 ± 12.8 [47]	120.0 ± 14.0 [43, 48]	115.7 ± 12.7 [43]	114.7 ± 12.5 [44]	117.0 ± 13.2 [45]	116.0 ± 12.5 [38]	117.5 ± 11.8 [39]
	**TD**	3058 ± 433 [105]	3058 ± 433 [105]	3058 ± 433 [105]	2840 ± 630 [46]	2960 ± 660 [47]	2990 ± 640 [43, 48]	2940 ± 590 [43]	2940 ± 610 [44]	3000 ± 540 [45]	3040 ± 570 [38]	3140 ± 530 [39]

*Q1* quarter 1, *Q2* quarter 2, *Q3* quarter 3, *Q4* quarter 4, *FWD* forward, *NOM* nomadic, *DEF* defender, *TD* total distance (m), *m.min*^*-1*^ metres per minute, *TMT* total match time (min), *TO* time over 18 km·h^-1^ (min)

### Meta-regression

**Fig. 2:**
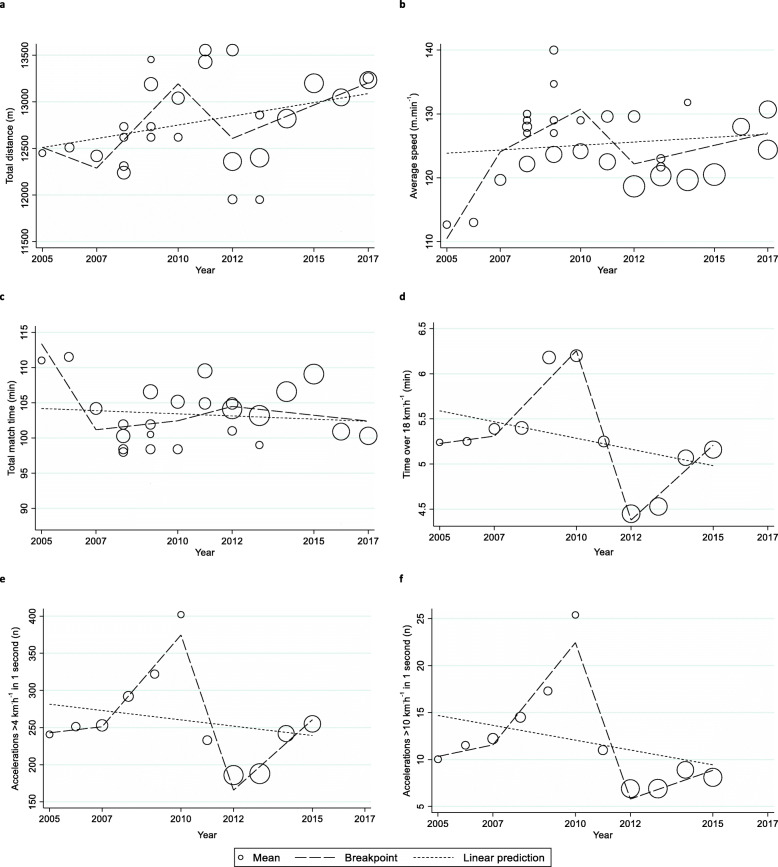
Regression analysis of the relationship between year (*x* axis) and **a** total distance, **b** average speed, **c** total match time, **d** time over 18 km·h^−1^, **e** number of accelerations greater than 4 km·h^−1^ in 1 s, and **f** number of accelerations greater than 10 km·h^−1^ in 1 s (*y* axis). *n* count of accelerations. The size of the circle is representative of the sample size of the data point

There was uniformity in the reporting of GPS metrics for overall total distance and m.min^-1^ for quarters and positional groups, overall match duration and time over 18 km·h^-1^ for positional groups and overall accelerations greater than 4 km·h^-1^ and 10 km·h^-1^ in one second. This resulted in 28 studies [2, 8, 17, 22, 24, 36, 38–48, 105–115] being included in the meta-regression as this allowed volume and intensity to be analysed over time. Table 3 summarises the mean change per year in each metric, within seasonal periods of interest (e.g. 2005 to 2007 breakpoint), with the *p* value representing the level of significance of the slope of the regression. Regression analysis of each GPS metric is provided in Figure 2.

**Table 3:** Data extraction from studies included in meta-regression (data are mean change per year ± 95% CI)

	Overall (2005–2017)	2005–2007	2007–2010	2010–2012	2012–2015	2012–2017
**Total distance (m)**						
Overall	48 ± 57, *p* < 0.094	− 111 ± 535, *p* < 0.670	302 ± 285, *p* < 0.039	− 293 ± 316, *p* < 0.068	-	119 ± 141, *p* < 0.092
Forward	36 ± 95, *p* < 0.416	− 234 ± 735, *p <* 0.476	399 ± 364, *p* < 0.036	− 521 ± 459, *p* < 0.032	-	240 ± 311, *p* < 0.111
Nomadic	10 ± 127, *p* < 0.864	− 299 ± 1020, *p* < 0.500	353 ± 438, *p* < 0.097	− 541 ± 579, *p* < 0.062	-	265 ± 429, *p* < 0.182
Defender	95 ± 122, *p* < 0.111	− 274 ± 677, *p* < 0.370	710 ± 383, *p* < 0.003	− 686 ± 503, *p* < 0.015	-	236 ± 338, *p* < 0.143
Quarter 1	− 7 ± 30, *p* < 0.616	− 90 ± 202, *p* < 0.317	− 8 ± 125, *p* < 0.887	− 56 ± 173, *p* < 0.457	-	91 ± 125, *p* < 0.125
Quarter 2	16 ± 25, *p* < 0.174	− 66 ± 160, *p* < 0.353	25 ± 96, *p* < 0.553	− 39 ± 127, *p* < 0.477	-	106 ± 91, *p* < 0.029
Quarter 3	− 12 ± 24, *p* < 0.275	− 83 ± 187, *p* < 0.321	− 24 ± 114, *p* < 0.624	6 ± 155, *p* < 0.924	-	18 ± 111, *p* < 0.706
Quarter 4	6.0 ± 19, *p* < 0.494	− 63 ± 122, *p* < 0.253	0 ± 70, *p* < 0.989	− 11 ± 88, *p* < 0.780	-	65 ± 62, *p* < 0.045
**Metres per minute**						
Overall	0.2 ± 0.7, *p* < 0.494	6.8 ± 5.8, *p* < 0.023	2.2 ± 3.1, *p* < 0.155	− 4.3 ± 3.6, *p* < 0.022	-	1.0 ± 1.7, *p* < 0.243
Forward	0.4 ± 0.6, *p* < 0.185	1.5 ± 4.3, *p* < 0.477	2.2 ± 1.8, *p* < 0.021	− 2.8 ± 1.8, *p* < 0.007	-	0.6 ± 0.8, *p* < 0.108
Nomadic	0.7 ± 1.4, *p* < 0.275	2.5 ± 10.9, *p* < 0.617	2.3 ± 6.8, *p* < 0.448	− 2.2 ± 9.2, *p* < 0.615	-	1.0 ± 6.5, *p* < 0.729
Defender	0.7 ± 1.1, *p* < 0.211	0.4 ± 7.9, *p* < 0.912	4.4 ± 4.9, *p* < 0.072	− 3.6 ± 6.5, *p* < 0.235	-	0.3 ± 4.8, *p* < 0.892
Quarter 1	0.7 ± 0.9, *p* < 0.106	2.3 ± 4.5, *p* < 0.269	3.2 ± 2.7, *p* < 0.026	− 3.8 ± 3.5, *p* < 0.034	-	1.2 ± 2.5, *p* < 0.300
Quarter 2	1.2 ± 1.2, *p* < 0.045	2.8 ± 5.8, *p* < 0.286	5.0 ± 3.50, *p* < 0.013	− 4.4 ± 4.7, *p* < 0.063	-	0.9 ± 3.4, *p* < 0.559
Quarter 3	1.1 ± 1.2, *p* < 0.071	2.8 ± 5.4, *p* < 0.246	4.7 ± 3.2, *p* < 0.011	− 4.4 ± 4.1, *p* < 0.038	-	0.7 ± 3.0, *p* < 0.607
Quarter 4	1.4 ± 1.2, *p* < 0.026	2.8 ± 6.1, *p* < 0.298	4.8 ± 3.8, *p* < 0.019	− 3.9 ± 5.0, *p* < 0.108	-	1.1 ± 3.7, *p* < 0.481
**Total match time (min)**						
Overall	− 0.15 ± 0.57, *p* < 0.591	− 6.10 ± 4.47, *p* < 0.010	0.42 ± 2.37, *p* < 0.714	1.02 ± 2.71, *p* < 0.443	-	− 0.41 ± 1.37, *p* < 0.536
Forward	− 0.15 ± 0.73, *p* < 0.646	− 2.67 ± 6.77, *p* < 0.382	0.76 ± 3.53, *p* < 0.625	− 1.87 ± 4.59, *p* < 0.365	-	1.34 ± 3.28, *p* < 0.366
Nomadic	0.02 ± 0.96, *p* < 0.958	− 4.97 ± 8.83, *p* < 0.217	1.59 ± 3.78, *p* < 0.343	− 2.38 ± 4.99, *p* < 0.286	-	1.63 ± 3.72, *p* < 0.323
Defender	0.10 ± 0.92, *p* < 0.799	− 2.94 ± 8.36, *p* < 0.432	1.61 ± 4.84, *p* < 0.457	− 1.85 ± 6.49, *p* < 0.521	-	1.35 ± 4.66, *p* < 0.513
**Time over 18 km·h**^**−1**^ **(min)**						
Overall	− 0.06 ± 0.11, *p* < 0.271	0.04 ± 0.31, *p* < 0.761	0.31 ± 0.17, *p* < 0.005	− 0.93 ± 0.25, *p* < 0.001	0.27 ± 0.18, *p* < 0.008	-
Forward	− 0.06 ± 0.08, *p* < 0.096	− 0.15 ± 0.53, *p* < 0.515	0.18 ± 0.26, *p* < 0.144	− 0.55 ± 0.34, *p* < 0.007	0.13 ± 0.22, *p* < 0.196	-
Nomadic	− 0.70 ± 0.12, *p* < 0.238	− 0.04 ± 0.56, *p* < 0.845	0.29 ± 0.23, *p* < 0.020	− 0.84 ± 0.31, *p* < 0.001	0.26 ± 0.22, *p* < 0.029	-
Defender	− 0.09 ± 0.15, *p* < 0.208	− 0.41 ± 0.83, *p* < 0.273	0.48 ± 0.52, *p* < 0.062	− 1.09 ± 0.72, *p* < 0.010	0.29 ± 0.51, *p* < 0.207	-
**Accelerations greater than 4 km·h**^**−1**^ **in 1 s (*n*)**						
Overall	− 4.2 ± 13.5, *p* < 0.498	4.1 ± 35.4, *p* < 0.788	41.1 ± 22.5, *p* < 0.004	− 104.4 ± 32.2, *p* < 0.001	31.6 ± 23.1, *p* < 0.016	-
**Accelerations greater than 10 km·h**^**−1**^ **in 1 s (*n*)**						
Overall	− 0.5 ± 1.2, *p* < 0.335	0.6 ± 3.2, *p* < 0.658	3.6 ± 2.1, *p* < 0.005	− 8.3 ± 2.9, *p* < 0.001	1.0 ± 2.1, *p* < 0.281	-

#### Total distance

Total distance was unchanged from 2005 to 2017 (*p* = 0.094; Table 3). However, between the 2007 to 2010 seasons, total distance increased across the AFL (*p* = 0.039; Figure 2a) and for forwards (*p* = 0.036; Table 3) and defenders (*p* = 0.003; Table 3).

Across the 2010 to 2012 seasons, total distance decreased for forward (*p* = 0.032; Table 3) and defensive (*p* = 0.015; Table 3) positional groups. Increases in total distance were observed across second (*p* = 0.029; Table 3) and fourth (*p* = 0.045; Table 3) quarters from 2012 to 2017.

#### Metres per minute

There was no change in metres per minute across all matches from 2005 to 2017 (*p* = 0.494; Table 3). However, m.min^−1^ increased in the second (*p* = 0.045; Table 3) and fourth (*p* = 0.026; Table 3) quarters during this period.

From 2005 to 2007, m.min^−1^ increased across the AFL (*p* = 0.023; Fig. 2b), with significant increases recorded across all four quarters from 2007 to 2010 (Table 3). From 2010 to 2012, m.min^−1^ decreased (*p* = 0.022; Fig. 2b), particularly in the first (*p* = 0.034; Table 3) and third (*p* = 0.038; Table 3) quarters.

There was no change in metres per minute from 2012 to 2017 (*p* = 0.243; Table 3).

#### Total match time

No change in match duration was found from 2005 to 2017 (*p* = 0.591; Table 3). However, there was a significant decrease in match duration from 2005 to 2007 (*p* = 0.010; Fig. 2c).

#### Time over 18 km·h^−1^

There was no change in the amount of time players spent over 18 km·h^−1^ from 2005 to 2015 (*p* = 0.271, Table 3). Competition wide increases were recorded across the 2007 to 2010 (*p* = 0.005; Fig. 2d) and 2012 to 2015 (*p* = 0.008; Fig. 2d) periods. Nomadic players were the only positional group to record significant increases across both periods (Table 3).

From 2010 to 2012, time spent over 18 km·h^−1^ decreased across the competition (*p* < 0.001; Fig. 2d) and for all positional groups (Table 3).

#### Accelerations greater than 4 km·h^−1^ in 1 s

There was no change in the number of accelerations greater than 4 km·h^−1^ in 1 s from 2005 to 2015 (*p* = 0.498, Table 3). The number of accelerations increased from 2007 to 2010 (*p* = 0.004; Fig. 2e) and from 2012 to 2015 (*p* = 0.016, Fig. 2e). However, across the 2010 to 2012 seasons, the number of accelerations decreased (*p* < 0.001, Fig. 2e).

#### Accelerations greater than 10 km·h^−1^ in 1 s

The number of accelerations greater than 10 km·h^−1^ in 1 s was unchanged from 2005 to 2015 (*p* = 0.335, Table 3). The number of accelerations increased from 2007 to 2010 (*p* = 0.005, Fig. 2f) but decreased from 2010 to 2012 (*p* < 0.001, Fig. 2f).

## Discussion

This systematic review sought to quantify shifts in the physical match demands (volume and intensity) of AFL players using GPS match data. Since 2005, when clubs were first permitted to use GPS technology to monitor the in-game movements of players, studies have investigated and reported on various GPS outputs such as total distance, average speed, match duration, time spent over certain speeds, and the number of accelerations. To the knowledge of the authors, no peer-reviewed study has examined the longitudinal relationship of these metrics in quantifying the evolution of AFL match demands using GPS technology.

The primary findings were that the volume and intensity of match demands of elite AF players remained relatively stable between 2005 and 2017. However, within this time frame there were shifts in volume and intensity defined predominately by evolutionary changes across discrete seasonal periods. AF is a complex game with many variables, and evolutionary changes in player activity profiles are likely attributable to a combination of factors: rule changes (including umpiring interpretations) [116], player attributes (improved fitness and professionalism of players and the sport [116, 117], including sports science staff) and game style tactics (coaching strategies) [15].

Six GPS derived measures of volume and intensity were reported with the requisite level of consistency to warrant inclusion in the meta-regression: total distance, player movement speed in metres per minute, match duration, time over 18 km·h^−1^, and accelerations greater than 4 km·h^−1^ and 10 km·h^−1^ in 1 s, respectively. These measures are discussed below in the context of the discrete evolutionary periods identified in the breakpoint analysis from the meta-regression.

### 2005–2007

This review found a reduction in volume from 2005 to 2007, evidenced by a decrease in average match time of 6.10 minutes per season during this period (Table 3; Figure 2c). The reduction in player match time is supported by smaller, observational studies conducted over finite periods prior to 2010, which also observed similar decreases [17, 22, 46]. However, an increase in player average movement speed of 6.8 m.min^-1^ per season from 2005 to 2007 (Table 3; Figure 2b), suggests an increase in intensity of AFL matches. This period coincided with a rapid increase in the use of the interchange bench to facilitate player recovery, evidenced by an increase of 37 rotations in 2005, to 92 per team per game in 2009 [116]. Teams also began to develop a more comprehensive understanding of their players’ match activity profiles with the introduction of GPS analysis to the AFL in 2005.

The AFL introduced a number of rule and rule interpretation changes in 2005 and 2006 to increase the speed of the game, promote more continuous play and ultimately restore the traditional aspects of the game [116]. For example, players were afforded less time to dispose of the ball after a mark or free kick, along with a stricter interpretation of deliberate out of bounds and quicker boundary throw-ins [116]. These changes influenced the style of play and movement demands of the players. Play periods were more continuous (average of 35 – 45 seconds), ball movement was faster and longer, there were more running bounces, and play time in the game increased from about 50 to 60% of total match time [116]. The combined impact of rule changes and increase in interchange rotations during this period is a likely contributor and explanation to the reduction in match time (volume) and the associated increased game intensity (m.min^-1^).

Woods et al. [15] suggest from 2005 to 2009, game style in the AFL focused on maintaining possession of the ball and controlling the ‘tempo’ of the match, characterised by frequent passing resulting in significant increases in the number of disposals and uncontested possessions. Earlier studies [27, 57, 106] have established that greater average speed resulted in more technical involvement (possessions) throughout a match. Therefore, this review’s findings of an increase in intensity from 2005 to 2007, quantified as m.min^-1^, supports the association in the literature between increased player average movement speed, number of possessions and the widely adopted game style around this time. However, the increase in the number of possessions is confounded by the increase in proportion of play time in which the ball was ‘in play’, from just less than 50% to almost 70% across this period [118].

In summary, the reasons for the increased intensity and reduced volume from 2005 to 2007 are multifactorial, and potentially best explained by increases in ball movement speed [116], offensive play [118], utilisation of the interchange bench [118] and the requirement for players to work harder offensively to maintain possession of the ball.

### 2007–2010

In each season from 2007 to 2010, total distance increased by 302 m (Fig. 2a) and by 399 m and 710 m each year for forwards and defenders respectively (Table 3). Average player movement speed also increased across all four quarters during this period (Table 3), with forwards recording an increase of 2.2 m.min^−1^ each year (Table 3).

This review also found increases in the amount of time players ran at speeds exceeding 18 km·h^-1^ (Figure 2d), with nomadic players the only positional group to record an increase during this period (Table 3). This is consistent with findings from a smaller cohort study comparing the 2003 and 2009 seasons, which found increases in the percentage of time players spent sprinting at speeds in excess of 20 km·h^-1^ [17], albeit with some measures using different technologies. Similarly, the number of accelerations greater than 4 km·h^-1^ and 10 km·h^-1^ in one second also increased across the AFL (Table 3; Figure 2). The latter finding is supported by earlier research [114] within this period, which found substantial increases in the number of maximal accelerations performed by elite AF players across the 2008 to 2009 seasons.

These findings potentially suggest the AFL’s rule changes in 2005 and 2006, implemented with the intention of decreasing the duration of stoppages to make the game more continuous by fatiguing players [13, 116], are one contributor to the resultant increases in the volume and total distance from 2007 to 2010. To counteract the increased player fatigue and resultant increase of game-demands on players, a rapid increase in the use of the interchange bench ensued, from approximately 37 in 2005 to 119 per team per game in 2010 [22, 28, 29]. This led to rested players re-entering the field in a state of ‘freshness’, producing greater movement speeds [22, 28, 29] and a shift in game style [15]. The spike in interchange rotations during this time is a likely contributor to the increase in high-intensity efforts such as the number of accelerations and volume of high-speed running (speed greater than 18 km·h^-1^) found by this review, which is supported by earlier research [22].

The evolution of game style during this time [72] coincides with a finding of this review of an increase in forward line players’ average speed from 2007 to 2010. This is likely due to a forward’s requirement to push further up the ground to create options to maintain possession, or impact defensive pressure and then work back to the forward line to create an attacking option when required.

The evolution of a possession-based game style is also consistent with, from 2007, a rapid increase in defensive play patterns, combined with a decrease in offensive play [118]. Such changes in game style are characterised by increases in defensive statistics such as stoppages, tackles [15] and congestion [116]. For example, during the period from 2008 to 2010 ball movement distance, play period duration and percentage play time all decreased significantly as the number of stoppages increased, while player density began to accelerate [116]. This seems contradictory to the finding of increased player movement distance found in this review. One explanation might lie in the coaching strategies of the day where instructions to clear the frequent congestion involved ‘rapid spread’ to create passing options. This combination of slow, congested play combined with quick movement from the high-density regions, may explain the relatively constant average movement speed (m.min^-1^) and increases in the number of accelerations and high-speed running time (time over 18 km·h^-1^ ) found in this review during these seasons.

Since increased congestion affords players less time and space for skill execution and decision making [118], the increase in acceleration counts during this period is likely to be reflective of the need for players to accelerate in and out of space to create and cover offensive and defensive options. This inference is supported by Johnston et al. [107] who suggest an increased number of technical involvements is indicative of players being in a confined space, leading to greater physical contact, change of direction and number of accelerations.

### 2010–2012

From 2010 to 2012, the volume of match demands appeared to stabilise despite respective decreases in the distance covered by forward and defensive players of 521 and 686 m per season (Table 3). This is in the context of a competition wide reduction in intensity, quantified by decreases in metres per minute, high-speed running (greater than 18 km·h^−1^) and the number of accelerations (Fig. 2; Table 3).

Following increases in the volume and intensity of game demands from 2007 to 2010, the rapid increase of defensive play [118], associated player congestion [116] and the accelerating rate of interchange rotations [116], the AFL implemented a number of rule changes ahead of the 2011 season. Most notably, the number of players available for rotation was limited to three, in addition to a substitute player who could be used at a team’s discretion. Further changes included a stricter interpretation of the deliberate out of bounds rule and infringed players granted the liberty to determine when to take ‘advantage’ (to continue play after a free kick is awarded to their team). Game style also evolved from 2010, with Woods et al. [15] suggesting a collective game-plan emphasising re-possession of the ball through the implementation of full-ground zones. This strategy led to even more congestion and is characterised by a decline in handballs, disposals and uncontested possession, and an increase in contested possession, tackles and turnovers.

These changes, when considered in the context of a relative stabilisation of volume and reduction in intensity through decreases in m.min^-1^, high-speed running and the number of accelerations, are suggestive of game demands reflecting steady state running. It is therefore not surprising that with increased congestion, player density [116] and defensive play [118], players exhibited reductions in high-speed running data and the number of accelerations due to reduced space and time. Additionally, earlier research [29] has established coaches prioritise the use of the interchange bench for nomadic players given they experience greater physical match demands [22, 106]. Therefore, decreases in total distance for forward and defensive players during this period, is likely the product of changes in game style tactics rather than rule and interpretation changes.

Whilst the cause and effect relationship of a reduction in intensity and confounding game style tactics and rule changes are undoubtedly complex, the combination of rule, interpretation and game style changes [15], the overall emphasis on defensiveness, full-ground zoning and increased congestion appear the dominant factors. This is particularly the case with interchange rotation numbers increasing in 2012 and 2013 to record highs [116]. While higher rotations have been shown to be associated with an increase in intensity for individual players [29], this did not occur overall because of the defensive game strategies and associated congested play. This is despite the ‘substitute’ rule and modification of umpire interpretations designed to limit its effect.

### 2012–2017

Apart from respective increases in total distance in the second (106 metres) and fourth (65 metres) quarters each season from 2012 to 2017 (Table 3), there was no change in the volume of overall match demands in the AFL during this period. However, increased distance during the second and fourth quarters is potentially attributable to improvements in player professionalism and fitness [116]. Furthermore, increases in the duration of time spent above speeds of 18 km·h^-1^ and the number of accelerations greater than 4 km·h^-1^ in one second from 2012 to 2015, are indicative of an increase in intensity.

During the early stages of this period (2012 to 2013), the pattern of game-play remained orientated around defence whilst offensive play continued to decrease [118], with player density and congestion, stoppage time and number and tackle numbers increasing, and the percentage of play time and scoring decreasing [116]. These trends all occurred in the face of the ‘substitute’ rule from 2011, with interchange rotation numbers peaking at 133 on average per team per game in 2013 [116]. This precipitated the AFL’s decision to cap interchange rotations at 120 in 2014 [116], whilst still maintaining the ‘substitute’ rule.

As the interchange cap had minimal impact on player running loads [39], the increase in intensity found in this review, is perhaps best explained by Woods et al’s [15] suggestion that from 2014 coaches adopted a blended game style of possession and re-possession, effectively blending a unique combination of previously dominant game style tactics. This modified game style, where congestion, stoppages and slower ball speed were dominant facets of the game, is indicative of increases in high-speed running and accelerations requiring players to break into the space and away from congestion when able to do so [107]. Indeed, improved player professionalism and physical fitness are likely to have also been contributing factors. This inference is also potentially supported by further rule changes in 2013 whereby umpires began throwing the ball up for all field stoppages [116], eradicating the laborious and time consuming set up involved with bouncing the ball which often increased congestion around stoppages.

In 2016 the AFL decided to further cap interchange rotations to 90 and repeal the ‘substitute’ rule, restoring the number of interchangeable players to four, as well as introducing a protection zone of 10 metres for a player with a mark or free kick. Additionally, the growing trend towards the margin for teams adopting greater defensive play, compared to offensive play, continued to widen [118]. Despite these rule changes and game-play trends, in which it might be expected changes in intensity and volume would ensue, no such changes were found in this review.

### Standardisation of data

Data extracted from studies included in this review highlights a lack of uniformity in the reporting of GPS data from AFL matches in the literature (Table 1), which limited the number of studies eligible for inclusion in the meta-regression. The findings of this review highlights the need for consistency in the reporting of GPS data from AFL matches, which supports earlier recommendations of the need for universally accepted and standardised speed zones for GPS monitoring in team sport athletes [119] and the classification of magnitudes of accelerations [9]. Given the high degree of variability in high-speed running metrics [4], the need for standardisation is evidenced by the diversity in how such metrics are reported, as detailed in Table 1. This review supports standardisation of recording and reporting high-speed running data, as speeds greater than 4 m·s^-1^ or 14.4 km·h^-1^, which is consistent with the majority of AF studies reporting this metric [2, 24, 36, 104, 105, 112, 113].

## Limitations

As evidenced by the data summarised in Table 1, this review is limited by the amount of data reported by eligible studies, particularly those eligible for inclusion in the meta-regression. It is acknowledged that some studies analysed changes over multiple seasons, and therefore reported GPS data as the mean over 2- or 3-year periods, rather than reporting the mean value from individual seasons. Furthermore, the consistency and uniformity of data reporting, particularly in relation to high-speed running, limited the extent and detail of analysis that was possible in this review. This limitation is supportive of a recommendation for standardising and reporting of GPS data from AFL matches.

Additionally, in the absence of standardisation across AFL clubs in the manufacturer and sampling frequency of the GPS units used to collect and report on data, there is likely a level of inconsistency in the data reported by studies included in this review. Despite improvements in the reliability and validity of data collected from GPS units over time, it has been recommended comparisons should not be drawn between movement data collected from 10Hz Catapult MinimaxX and 15Hz GPSports GPS units [33] given validity and reliability issues associated with intermittent, high-speed activity. However, the combined use of acceleration and speed data elicits a more holistic representation of players’ physical demands [5], therefore excluding studies which reported data from either GPS unit, would reduce the comprehensiveness of the findings in this review.

This literature review is also limited by the anonymity of the source of data (i.e. AFL club[s]) reported by studies. Whilst this raises potential issues about the validity of some findings, wherever possible, the authors ensured duplication of data was avoided.

## Future research direction

Whilst this review has examined the evolutionary change in AF player match demands using GPS data, future research should look at using accelerometry data to supplement the findings of this review to provide a holistic assessment of changes in players’ activity profiles. Future research could examine changes in AFL GPS training data in conjunction with changes in the rate and nature of injuries, to analyse if any changes are reflective of the shift in match demands highlighted by this review. Furthermore, given the change to match demands found in this review, future research could analyse changes in fitness performance data of players such as sprints and repeated sprints across playing position, to examine whether the fitness and speed of players changed concomitantly. Finally, analysis of GPS data from the 2018, 2019, and 2020 AFL seasons would provide a more current reflection of the shift in modern game demands, particularly given the reduction of match duration in the 2020 season.

## Conclusion

This systematic literature review sought to quantify the change in volume and intensity related match demands of elite, AF players using GPS data from 2005 to 2017. In examining this relationship, studies reporting the requisite level of data from six GPS derived metrics were analysed. However, given the inconsistencies in the reporting of GPS data in AF matches, it is recommended a standardised and uniform method of recording and reporting GPS match data be developed.

No significant longitudinal changes were found in relation to total distance, m.min^−1^, total match time, time spent over 18 km·h^−1^ and the number of accelerations performed greater than 4 km·h^−1^ and 10 km·h^−1^ in 1 s. This review found changes in the volume and intensity of the match demands of AFL players were predominately defined by discrete evolutionary periods from 2007 to 2010 and 2010 to 2012. It is postulated rule changes and an evolving game style appear to have been the most prolific factors in the evolution of the match demands of elite AF players.

## Data Availability

All data generated or analysed during this study are included in this published article.

## Abbreviations

AFL: Australian Football League
AF: Australian rules football
CI: confidence interval
DEF: defender
FWD: forward
GPS: global positioning system
HIE: high-intensity efforts
HIR: high-intensity running
HSR: high-speed running
Hz: hertz
km^.^h^-1^: kilometres per hour
m: metres
min: minutes
m.min^-1^: metres per minute
m^.^s^-1^: metres per second
NOM: nomadic
PRISMA: Preferred Reporting Items for Systematic Reviews and Meta-Analyses
Q1: quarter 1
Q2: quarter 2
Q3: quarter 3
Q4: quarter 4
s: seconds
TD: total distance
TMT: total match time
TO: time over 18 km·h^−1^
